# How Healthy Is State Mental Health System in Madhya Pradesh, India? An Assessment of Today to Plan for a Better Tomorrow

**DOI:** 10.1155/2021/6364321

**Published:** 2021-03-10

**Authors:** Arun M. Kokane, Abhijit P. Pakhare, Gopalkrishna Gururaj, Mathew Varghese, Vivek Benegal, Girish N. Rao, Banavaram Anniappan Arvind, Krishna Prasad M, Arun Mitra, Mukesh Shukla, Kriti Yadav, Sukanya Ray, Akash Ranjan, Rajni Chatterji, Pankaj Mittal

**Affiliations:** ^1^Department of Community and Family Medicine, All India Institute of Medical Sciences, Bhopal, India; ^2^Department of Epidemiology, Centre for Public Health, National Institute of Mental Health and Neuro Sciences (NIMHANS), Bengaluru, Karnataka, India; ^3^Department of Psychiatry, National Institute of Mental Health and Neuro Sciences (NIMHANS), Bengaluru, Karnataka, India; ^4^Department of Psychiatry, Centre for Addiction Medicine, National Institute of Mental Health and Neuro Sciences (NIMHANS), Bengaluru, Karnataka, India; ^5^Department of Psychiatry, All India Institute of Medical Sciences, Bhopal, India; ^6^Department of Psychiatry, Bhopal Memorial Hospital and Research Centre (BMHRC), Bhopal, India

## Abstract

**Background:**

Mental illness affects over one-third of the Indian population, and only a little is known about the exact situation of health systems in Madhya Pradesh, India. Therefore, the present research work provides an assessment of state mental health systems in Madhya Pradesh.

**Methods:**

The present cross-sectional study was conducted as a part of National Mental Health Survey 2015-16 in 48 districts of Madhya Pradesh, to provide an overview of the status of mental health systems. Secondary data was also retrieved from the state office so as to present the situational analysis in a more comprehensive and inferential way. The proforma for the study was developed based on the experience gained from studies conducted earlier with World Health Organization's Assessment Instrument for Mental Health Systems (WHO-AIMS) and with WHO's Mental Health Atlas as the base for thematic analysis.

**Results:**

Out of 51 districts, 13.7% of the districts of the state have been covered under District Mental Health Program (DMHP) in 2015-16. Around 11.8% of district/general hospitals were involved in providing mental health services. The availability of psychiatrist was 0.05 per Lakh population. Around 0.2% of the total health budget was allocated by the state for the last financial year for mental health. The overall average score of Madhya Pradesh in the assessment of qualitative indicators was 31 out of 100 in the year 2015-16.

**Conclusion:**

There is huge scope and an urgent need to increase mental healthcare facilities (with upgradation of existing one) along the availability of mental healthcare staff.

## 1. Introduction

Over 450 million people are expected to be suffering from neuropsychiatric conditions worldwide. There has been a substantial increase over the past few decades in the proportionate total Disability Adjusted Life Years (DALYs) lost due to all illnesses and injuries accountable to mental and neurological disorders. It has increased from 10% in 1990 to 12% in 2000 and is expected to further increase to 15% by the year 2020 [1]. This increasing burden will further increase the treatment gap to 75% in low- and lower-middle-class income countries like India, where the resources are limited and poorly utilized [2].

The emphasis on mental health has been laid in the World Health Report (2001) which called for increased and focused efforts towards the strengthening of the mental health care system [3]. A comprehensive, robust, and effective health system where all stakeholders actively involve through intersectoral coordination and community participation to promote, restore, and sustain health [4, 5].

The etiology and forthcoming consequences of mental illness are extremely complicated and need to be tackled collectively across the healthcare system rather than in isolation [6]. Considering the huge burden of mental health problems and its treatment gap, a well-organized and robust mental health system is crucial for tackling the problem. This system must encompass all the activities whose primary purpose is to promote, restore, or maintain mental health.

As mental disorders are becoming more prevalent globally, health care providers would require reliable information about the current status of mental health system. It would help delineate specific needs of the population and formulate specific strategies for addressing these pertinent issues in a more holistic manner which would eventually reduce the disparities [7]. Thus, the system approach in the mental health used in the present study will facilitate policy makers and implementers to formulate a basic framework through which high-quality mental health services could be delivered so as to reduce the gap of demand and supply in context to mental health and boost preventive measures along with the due implementation of rehabilitative services. Assessment of preparedness of mental health system at the district level would facilitate policymakers in prioritizing key domains for improvement. With this background, the current research paper presents a comprehensive and systematic evaluation of various components and subcomponents of health systems, at each level, that are involved in the provision of mental health services in Madhya Pradesh. It would help elucidate the challenges it poses to health care systems and recommend the way forward for revamping the health care systems around the world for improved quality assessment which would ultimately improve the quality of mental health care [7].

The study holds relevance at the global level also as there is dearth of evidence regarding mental health systems across the LMICs, UMICs, and HICs worldwide, with the last large-scale survey being done in 2007-09 [8, 9]. As there is paucity of research on this domain especially in context of developing nations where the condition of mental health delivery system is deplorable, therefore, the findings and recommendations of the current study would be of paramount importance in shaping the mental health delivery not only in our country, but it will pave way for further such studies and interventions in other developing nations of the world also.

## 2. Material and Methods

This study was conducted as a part of a National Mental Health Survey 2015-16 for the state of Madhya Pradesh, India.

### 2.1. Study Design

The status of state mental health was assessed using a mixed approach (quantitative and qualitative) on preselected parameters. Initially, an overview of general information related to population characteristics was gathered. These all adopted characteristics formed the overall base and were closely associated with and likely to influence the mental health of the state [10].

### 2.2. Study Period

Information related to State Mental Health System was collected between October to December 2015 and therefore depicts the status of the system as of December 2015 only.

### 2.3. Study Settings

According to the 2011 census, Madhya Pradesh has a population of about 72,626,809. The main work force constitutes 31.2% of the population. Urban residents and tribal population constitute of about 27.6% and 21.1%, respectively [11]. The state of Madhya Pradesh is constituted of 51 districts of which 48 districts were included in this study. The data from the rest three districts was not provided by the district authorities. The 48 districts of Madhya Pradesh included *Dewas*, *Sehore*, *Shivpuri*, *Satna*, *Mandla*, *Chhindwara*, *Barwani*, *Ratlam*, *Guna*, *Damoh*, *Morena*, *Bhind*, *Sheopur*, *Gwalior*, *Datia*, *Tikamgarh*, *Chhatarpur*, *Panna*, *Ashok Nagar*, *Rewa*, *Sidhi*, *Shadol*, *Anuppur*, *Umaria*, *Katni*, *Jabalpur*, *Sagar*, *Vidisha*, *Rajgarh*, *Shajapur*, *Mandasaur*, *Neemuch*, *Bhopal*, *Raisen*, *Narsingpur*, *Seoni*, *Balaghat*, *Hoshangabad*, *Ujjain*, *Indore*, *Alirajpur*, *Jhabua*, *Dhar*, *Khargone*, *Khandwa*, *Burhanpur*, *Betul* and *Harda*.

### 2.4. Study Tool and Data Sources

The data collection tool was developed and finalized based on experience gained from studies conducted earlier in Kolar (Karnataka) and Tamil Nadu with WHO-AIMS and the WHO Mental Health Atlas as the base for thematic analysis [12, 13]. Secondary data was also retrieved from the state health system to present the situational analysis in a more inferential and ample way.

### 2.5. Data Collection

The questionnaire was mailed to all district nodal officers. Then, the Field Data Collector (FDC) went to each district to collect the data. The collected data was scrutinized and cleaned by the investigators and checked for errors and missing information by contacting the respective district nodal officer. Also, investigators themselves searched secondary data sources to collect information. After consultation with stakeholders and domain experts, the questionnaire-based proforma was constructed, and training was provided by experts to field level data collectors.

#### 2.5.1. Components of Assessment

The data collection tool basically consists often domains and subdomains assessing primarily the general information about the current status of state in context to health-related resources, i.e., governmental and nongovernmental healthcare institutions, availability of human resource, and state health management information systems (HMIS) along with evaluation of policy, action plan by the state authorities at grass root level. Apart from that other indirect indicators like conduction of routine mental health activities and legislation related to mental health, its implementation, financing, and budgetary provisions, availability of drugs, intra- and intersectoral collaboration, activities of social welfare, inclusion of civil societies in mental health programs, IEC activities, and monitoring were also explored through present study. Multiple sources and key stakeholders both inside and outside the health care system at all levels were actively involved at different stages.

#### 2.5.2. Domains of Assessment

Once the data sets were completed, a set quantitative indicator covering essential domains of the mental health system (coverage of district mental health program, mental health service, mental health care facilities, human resource for mental health, treatment gap, mental health financing, and suicide) was developed for assessment. Apart from that five morbidity indicators based on the current burden of mental illness were also used for assessment purpose.

#### 2.5.3. Qualitative Indicators

Also, a set of 10 qualitative indicators each assessed on a scale from 1 to 10, covering essential domains of mental health system (mental health policy, mental health action plan, state mental health coordination mechanism, budget and training program for mental health, availability of drugs, IEC materials and health education activities, intra- and intersectoral collaboration, monitoring, and implementation of state legislation), based on a scoring pattern has been developed for assessment purposes.

Further details of the study methodology are published elsewhere [14]. This article was written in accordance with the “Strengthening the reporting of observational studies in epidemiology (STROBE)” statement recommendations [15].

## 3. Results

The District Mental Health Program (DMHP) was launched under NMHP in the year 1996. In MP DMHP program started in 1997-98, it encompassed the following:

1. Integration of mental health care services
2. Utilization of the existing infrastructure
3. Work-oriented training to the currently deployed staff
4. Incorporation of mental health services within the existing community development programs

MannKaksha and counselling centres were established to provide the above-mentioned services [15].

### 3.1. Coverage of District Mental Health Program (DMHP)

**Figure 1:**
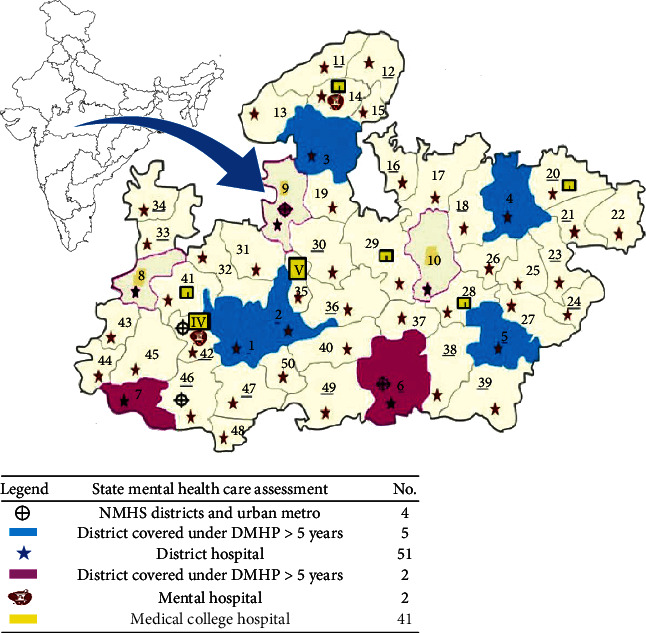
Map of Madhya Pradesh depicting health and mental health institutions in the state.

About 13.7% of the districts in the state have been covered under DMHP, serving about 14.2% of the general population and about 19.05% of the tribal population. There were only two districts where DMHP were started before 2012 (Table 1) District wise map of Madhya Pradesh showing mental health facilities and coverage by District Mental Health Program is shown in Figure 1.

#### 3.1.1. Mental Health Services

There were only 0.03 core government hospitals per 100000 population providing mental health facilities in the state. Around 1.18 beds were available for mental health in-patient services per 100000. Around 11.8% of district/general hospitals and 3.03% of the community health centers (CHCs) were involved in providing mental health services (Table 1).

#### 3.1.2. Mental Healthcare Facilities

There were only two mental hospitals (0.003 per 100,000population) and fourteen medical colleges with psychiatry units (0.019 per 100,000 population) providing mental health services. Six general hospitals have a designated psychiatry unit. There were no mobile mental health units, residential halfway centers, long-stay homes, hostels, or sheltered workshops in the state (Table 2). Although there were 148 health care professionals per 100,000 population available in the state, only 37 psychiatrists and 5 clinical psychologists and counsellors who provide mental health services were available for the same population (Table 3). Of all the health care professionals, only 99 (1 per million) underwent training in mental health in the last three years.

#### 3.1.3. Health Professionals

In the state of Madhya Pradesh, there were 99 nurses and 7 psychiatric social workers per 10 million population. Rehabilitation workers and special education teachers were virtually nonexistent (Table 3).

#### 3.1.4. Treatment Gap

The treatment gap in context to mental health services was observed to be present among 91% of people with mental illness, and these patients do not have any sort of health seeking for proper treatment. The number of suicides in the state was 1.19 per million population with the rate being highest among the 18-45 years age group.

#### 3.1.5. Mental Health Financing

A meager 0.2% of the total health budget was allocated by the state health department for the last financial year for mental health. However, the details regarding the exact utilization budget could not be assessed from authorities.

#### 3.1.6. Qualitative Assessment of State Mental Health System

The overall average score of Madhya Pradesh in the assessment of qualitative indicators was 31 out of 100. On the qualitative assessment of various domains of state mental health system, the state scored 7 out of 10 for both mental health coordination mechanism and availability of drugs. The state scored 5 out of 10 on the implementation status of the legislation. However, the state scored poorly in the rest of the domains like budget, training, and health education (Figure 1).

## 4. Discussion

The findings of this study provide valuable insights into the state of mental health care system in Madhya Pradesh. Just like the findings of previous studies, the distribution and availability of mental health services of Madhya Pradesh were found to be inequitable in distribution [16, 17] (Table 1).

As per state-level surveys, an estimated 6.6 million people are in need of mental health care [18]. The present study was itself a part of a collaborative National Mental Health Survey 2015-16 in Madhya Pradesh [19]. The District Mental Health Program (DMHP) has been the implementation underarm of the NMHP and has been an ongoing program since 1996 [20]. In Madhya Pradesh, about 13.7% of the districts of the state have been covered under DMHP, which is comparable to the estimate for the state of Punjab where 13.64% of the districts are covered with 14.9% population coverage. However, when compared with southern states like Kerala and Tamil Nadu where DMHP covers the whole population of all the districts, the disparity becomes evident [19] (Table 1). Similar findings have been noted on the international domain as documented in the WHO-AIMS report [8] (Table 4).

India's healthcare infrastructure as well as the delivery mode of the services is quite complex [21]. The private health sector comprised 8% of healthcare facilities in 1949 and has increased to 93% of the hospitals and 85% of the doctors by the end of 2012 [22]. The authors found that about 2.12 government and 0.25 private healthcare facilities were available per million population in Madhya Pradesh (Table 2). Though this estimate seems higher when compared to states like Uttar Pradesh where 1.48 health facilities are available per million population, it is much lower when compared to a state like Chhattisgarh where 4.65 health facilities were available [19]. Although government facilities outnumbered the private facilities, private practitioners still are the major health care providers in both urban and rural settings.

In order to provide good quality healthcare services, a well-trained and accountable workforce is critical. Also, unless there is substantial support from the community health workers, the medical officers alone would fail in the provision of quality healthcare. Similar to the finding of previous studies, the situation on human resources is quite alarming, although not surprising [16, 17]. Severe shortages of skilled mental health professionals in our country's health system and low level of mental health awareness and stigma against people with mental illness have ultimately resulted in seclusion of mental health from primary healthcare system [16].

In Madhya Pradesh, during the survey, it was found that about 12.4 healthcare workers were present per million population (Table 3). It was not only lower as compared to neighboring states like Uttar Pradesh where workforce density was around 19.3 per million but also lower compared to other states across India like Kerala, Manipur, Punjab, Rajasthan, and Tamil Nadu [19]. When the availability of doctors was visualized in Madhya Pradesh, it was almost equal to the other neighboring states like Chhattisgarh, Gujarat, Jharkhand, and Uttar Pradesh (5-6 per 1,00,000 population); on the other hand, it was lower when compared to the states like Kerala and Manipur [19] (Table 3). In respect to the skilled manpower, the number of psychiatrists in Madhya Pradesh are 5 times less than the recommended requirement of mental health professionals (i.e., Madhya Pradesh: 2 per million, recommended 10 per million) [23] (Table 3). This estimate is lower than the national average given by the World Mental Health Atlas 2014 for India, i.e., 3 per million [12]. The number of psychologists and psychiatric social workers was much lesser in Madhya Pradesh than the average national deficit of psychiatrists (77% deficit) [24] (Table 3). However, similar findings have also been reported globally [8, 20] (Table 5).

There were only few initiatives which made an attempt to enhance the capacity of health staff such as PHC medical officers and frontline health workers such as ANMs, Anganwadi workers, and ASHAs. There is strong evidence supporting the use of grass root level solutions with respect to mental health care [25–28]. Almost in all the states like Madhya Pradesh, the major proportion of the healthcare forces comprises frontline health workers like Auxiliary Nurse and Midwives (ANMs) and Accredited Social Health Activists (ASHAs) [19].

At the time of the study, there were two mental hospitals and fourteen medical colleges with dedicated psychiatry units in Madhya Pradesh (Table 2). This is in conjunction with the status of mental health care facilities across other states of India [19]. Besides these trained doctors, there is a need for supportive manpower also so as to deal with chronic and recurrent nature of these illnesses and to provide rehabilitative services if required.

**Figure 2:**
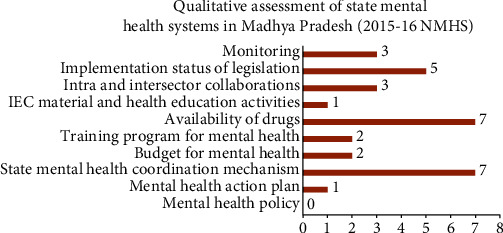
Qualitative assessment of state mental health system in Madhya Pradesh.

The existence of a mental health policy at national and state levels indicates a government's vision and intent for developing mental health services. In 2014, India unveiled its first National Mental Health Policy, which was accompanied by a mental health action plan for next one year. The policy was aimed at providing access to good quality treatment to mentally ill people, especially those living in poverty [29]. The policy places the responsibility of the decision and adoption of the National Mental Health Policy and integrates mental health with other policies of education, welfare, and housing. States like Gujarat and Kerala have adopted the policy, but at the time of survey (2015-16) Madhya Pradesh does not have any such stand-alone policy in place (Figure 2).

The Mental Health Act 1987 advocates for the establishment of a Mental Health Authority in each state to guide the governments on all issues associated with mental health [30]. To achieve a complete and efficient working mental health program, there is a need for a robust coordination mechanism among all stakeholders. However, such a mechanism was not visible absolutely in any of the states of India [19]. On the qualitative assessment of various domains of state mental health system in Madhya Pradesh, the average score for state mental health coordination mechanism was 7 out of 10 (Figure 2).

Almost all states including Madhya Pradesh have reported the implementation of the Indian Mental Healthcare Act, 2017 to some extent [19]. Madhya Pradesh scored 5 out of 10 in legislation domain (Figure 2). Healthcare financing indirectly reflects the government's intention on the mental health plans and policies. India's budget for mental health is less than 1% of the total health budget which itself has been criticized as being grossly inadequate. Also, at the state level, financing for mental health is a combination of funds from the central government, health and family welfare, and state contribution from the Dept. of Medical Education [19]. The utilization of the allocated budget was suboptimal and poor in Madhya Pradesh when compared to states like Gujarat and West Bengal where 71% and 83% of allocated funds gets utilized in due course [19] (Figure 2). Similar findings have been reported in LMICs and MICs worldwide [8, 31].

Information Education and Communication (IEC) activities on mental health were not conducted regularly in Madhya Pradesh. Only pamphlets and posters were used for these activities. States like Jharkhand, Uttar Pradesh, and Chhattisgarh also reported minimal IEC activities [19]. Regarding the availability of all essential drugs for mental healthcare, Madhya Pradesh scored 7 out of 10, which was similar to that of other states like Chhattisgarh, Assam, Gujarat, Jharkhand, and Rajasthan [19, 32] (Figure 2).

The global findings also corroborate with the findings of the present study. In a study done by Kilbourne et al., it is reported that mental disorders despite of being common worldwide had substandard quality of care which has not increased to the same extent as that for physical conditions [7]. Mental health outcomes need to be assessed more routinely, and measurement-centric care must become an integral part of the overall culture. More attention should be devoted for training and capacity building of the workforce for quality improvement. Also, in a study done by Samartzis and Talias, the systematic measurement and monitoring of indicators and the measurement and quantification of quality through them were reported to be the basis for evidence-based health policy for enhancing of the quality of mental health services [33]. In a study done by Saymah, in Gaza, it was proposed that priority must be given to mental health training and investments in human and organizational resources along with legislative support for achieving the desirable outcome [34].

As research-derived information about mental health system is imperative for establishing specific needs in a given setting which would thereby facilitate development of customized, culturally apt and cost-effective individual, and collective interventions best suited to their needs. It would also help in investigating their optimal implementation and functioning and would highlight the barriers which prevent these strategies from being implemented. As the capacity to undertake such research in low- and middle-income countries is extremely limited, this makes the findings of these studies very much relevant to the global settings [31].

### 4.1. Limitations

Study reports the facts during the survey period (2015-16) only; hence, no comments regarding status of health system assessment before and after the survey could be made. This facility assessment questionnaire was self-reported, and objective verification was not done; due to this in some districts, facilities might have been overreported. However, results of study reflect suboptimal facility conditions despite of self-reporting which indicate need for improvement.

## 5. Conclusions

This study highlights an extensive picture including major gaps and provides for essential evidences for formulating and reinforcing mental health-related services in addition to recognition of key domains of integration within the already existing system for improving mental health care in the country. Institution of a robust and effective health system infrastructure that coalesces mental health with the public health system is the ultimate need of the hour. The state has to embrace the mental health policy with well-demarcated and discrete goals and objectives with huge focus on the treatment along with rehabilitation services for those with mental illness. Also, there is a demand for creating an understanding of mental health among the community members. Training and equipping of the existing healthcare force (PHCs doctors, ANMs, and other community health workers) for mental health, increasing the mental health professional accessibility and availability, and fostering a public-private partnership with quality mental health services could be effectively used to fill the treatment gaps. Also, the promotion of research on mental health would help in delineating the barriers in mental illness health seeking as well as for developing specific strategies for addressing these barriers.

## Figures and Tables

**Table 1:** Coverage of District Mental Health Program and mental health services in Madhya Pradesh (2015-16).

Coverage of DMHP (District Mental Health Program)	
Number of districts where DMHP is started during the 12th plan period (2012 to 2017) in the state.	2
Number of districts where DMHP was started earlier to 2012 in the state.	5
Percentage of districts in the state covered by DMHP	13.7
Percentage of state population covered by DMHP	14.2
Percentage of tribal population covered by DMHP	19.05

Mental health services	
Number of core mental health-based hospital facilities in the state per 100000 population	0.03
Available number of beds for mental health inpatient services in the state per 100 000 population	1.18
Percentage of district/general hospitals in the state providing mental health services	11.8
Percentage of Taluka (CHCs) hospitals in the state providing mental health services	3.03
Percentage of PHCs in the state providing mental health services	0.1

**Table 2:** Mental healthcare facilities in Madhya Pradesh (2015-16).

Number of mental health facilities in the state per 100,000 population (for each category)	Per 100,000 population	Number available
Mental hospitals	0.003	2
Medical colleges with a psychiatric department	0.019	14
General hospitals with psychiatric units	0.008	6
Mobile mental health units	0	0
Day care centre	0.003	2
De-addiction units/centres	0.010	7
Residential half way homes	0	0
Long stay homes	0	0
Hostel (quarter stay homes)	0	0
Vocational training centres	0.003	2
Sheltered workshops	0	0

**Table 3:** Availability of mental health professional in Madhya Pradesh and the treatment gap (2015-16).

Availability of mental health professional	Per 100,000 population	Number available
Psychiatrists	0.05	37
Clinical psychologists and counsellors	0.01	5
Nurses trained in mental health/nurses with DPN qualification	0.14	99
Psychiatric social workers	0.01	7
Rehabilitation workers and special education teachers	0	0
Professional and paraprofessional psychosocial counsellors	0	0
Number of health professionals/personnel trained in or working for mental health in the state per 100 000 population	0.2	148

**Table 4:** WHO-AIMS report, percentage of disorders treated in mental health facilities, by country income group (median %).

	Substance use disorders	Schizophrenia	Mood disorders	Neurotic disorders	Personality disorders	Other
LICs (*n* = 8)	3	19	18	10	1	31
LMICs (*n* = 16)	4	19	19	24	3	21
UMICs (*n* = 3)	4	13	23	23	3	32
Total (*n* = 28)	4	19	19	20	2	25

**Table 5:** WHO-AIMS report, summary of indicators for mental health facilities, by country income group (median %).

Income group		LICs	LMICs	UMICs	Total
Number of mental hospitals per 100000 population	*n*	13	24	5	42
	Median	0	0.02	0.05	0.03
Total number of mental health professionals working in mental health facilities per 100000 population	*n*	11	21	5	27
	Median	1.4	6.0	24.1	6.0
Percentage of mental hospitals organizationally integrated with mental health outpatient facilities	*n*	11	20	5	36
	Median	100%	100%	80%	100%
Population served by 1 mental hospital	*n*	11	20	5	36
	Median	7600000	3349980	2060473	3349980
No of beds per 100000 population	*n*	13	24	5	42
	Median	0.86	6.55	18.02	5.94
Change in beds in the last 5 years (%)	*n*	11	20	5	36
	Median	1%	0%	-22%	0%
Percentage of beds for children and adolescents only	*n*	13	24	5	42
	Median	0%	0%	1%	0%
Percentage of child/adolescent patients	*n*	11	18	4	33
	Median	6%	3%	4%	4%
Percentage of women patients	*n*	10	18	4	32
	Median	38%	37%	41%	38%
Average no. of days spent in mental hospitals	*n*	9	19	3	31
	Median	42.1	64.5	72.9	61.1
Occupancy rate (%)	*n*	9	19	4	32
	Median	80%	71%	90%	80%
Percentage of patients staying less than 1 year (%)	*n*	8	16	4	28
	Median	93%	65%	39%	66%
Percentage of patients staying 1–4 years	*n*	8	16	4	28
	Median	4%	18%	4%	7%
Percentage of patients staying 5-10 years	*n*	8	16	4	28
	Median	2%	7%	10%	7%
Percentage of patients staying more than 10 years	*n*	8	16	4	28
	Median	2%	5%	45%	5%
Percentage of involuntary admissions	*n*	8	13	3	24
	Median	19%	40%	5%	36%

## Data Availability

De-identified data of mental health system assessment can be obtained from corresponding author upon reasonable request.
